# Supplementary material for: Patterns of Intron Gain and Loss in Fungi
Source: PLoS Biol. 2004 Nov 30;2(12):e422. doi: 10.1371/journal.pbio.0020422 (PMC532390; doi:10.1371/journal.pbio.0020422)
Supplement: Table S1 — Also available at http://genes.mit.edu/NielsenEtAl/. (4.3 MB ZIP). [file pbio.0020422.st001.zip › NielsenEtAl/html/493.html]

AN6558.1.NCU00419.1.MG06311.1.FG06937.1


```
 CLUSTAL W (1.82) Multiple Sequence Alignments - Introns Inserted


Sequence 1: NCU00419.1	156 aa
Sequence 2: FG06937.1	146 aa
Sequence 3: MG06311.1	151 aa
Sequence 4: AN6558.1	137 aa
Alignment Length: 163 aa
Number Identitical Residues: 60 aa
Alignment Score (without introns) 2822


MG06311.1 	MAPNSRARQAEAEAADAVMDDAPTSAQVEDEAQ-------DEFEEAEEEEEE--EQRVKM
NCU00419.1	MG--KITKFGD-EGEEVTMKETPTSHQPEAQDDVSMADAGEEGEEGDEEYEEGEGTKVRL
FG06937.1 	MP--ARSKEEQPQAEDTNMEDAPASAQPETND--------EEVED-EEEEEEIEAQRVRI
AN6558.1  	MP-----ASVHSQDNDQSMLDAQAQNQ----------------DHIDETLLE--QKRIIV
          	*         ..:  :  * :: :. *                :. :*   *    :: :

MG06311.1 	0LPGSTETAASFEFLKEGHTMGNALRYIIMKN2PEVELCAYAIPHPSEHKMNLRIQTYA1
NCU00419.1	0LPGSTPTAASFEFLDENHTLGNALRYVIMKN2PDVEFCAYAIPHPSEAKMNVRIQTYE1
FG06937.1 	0LPGSTDTAASFEFTDEGHTLGNALRYIIMKN2PDVEFCAYSIPHPSEPKMNIRIQTYS1
AN6558.1  	0LPGATDTAASFQFEGEGHTLGNALRFAIMKN2PEVEFCGYTIPHPSESKMNIRIQTYD1
          	 ***:* *****:*  *.**:*****: **** *:**:*.*:****** ***:*****  

MG06311.1 	TEGTTAIEALEKGLKDLQELCDVVTDKFTIARDDFAGRME--MDS-
NCU00419.1	GT--TAVEALRKGLRDIQDLCDVVSDKFLEARKDFTDKKA--AASA
FG06937.1 	G---TAVDALKKGLVDIQEVCDVVADEFWTKREAYNAEQG--IDR-
AN6558.1  	ST--TAVEALEKGLDTLMDLCDVVTDKFTAARDEFNAEQANRMES-
          	    **::**.***  : ::****:*:*   *. :  .  .
```
